# Advancing Non-Line-of-Sight Communication: A Comprehensive Review of State-of-the-Art Technologies and the Role of Energy Harvesting

**DOI:** 10.3390/s24144671

**Published:** 2024-07-18

**Authors:** Yasir Al-Ghafri, Hafiz M. Asif, Naser Tarhuni, Zia Nadir

**Affiliations:** Department of Electrical and Computer Engineering, College of Engineering, Sultan Qaboos University, Muscat 123, Oman; s132332@student.squ.edu.om (Y.A.-G.); tarhuni@squ.edu.om (N.T.); nadir@squ.edu.om (Z.N.)

**Keywords:** non-orthogonal multiple access, multiple input multiple output, energy harvesting, cooperative NOMA, intelligent reflecting surface, unmanned aerial vehicles

## Abstract

Enhancing spectral efficiency in non-line-of-sight (NLoS) environments is essential as 5G networks evolve, surpassing 4G systems with high information rates and minimal interference. Instead of relying on traditional Orthogonal Multiple Access (OMA) systems to tackle issues caused by NLoS, advanced wireless networks adopt innovative models like Non-Orthogonal Multiple Access (NOMA), cooperative relaying, Multiple Input Multiple Output (MIMO), and intelligent reflective surfaces (IRSs). Therefore, this study comprehensively analyzes these techniques for their potential to improve communication reliability and spectral efficiency in NLoS scenarios. Specifically, it encompasses an analysis of cooperative relaying strategies for their potential to improve reliability and spectral efficiency in NLoS environments through user cooperation. It also examines various MIMO configurations to address NLoS challenges via spatial diversity. Additionally, it investigates IRS settings, which can alter signal paths to enhance coverage and reduce interference and analyze the role of Unmanned Aerial Vehicles (UAVs) in establishing flexible communication infrastructure in difficult environments. This paper also surveys effective energy harvesting (EH) strategies that can be integrated with NOMA for efficient and reliable energy-communication networks. Our findings show that incorporating these technologies with NOMA not only enhances connectivity and spectral efficiency but also promotes a stable and environmentally sustainable data communication system.

## 1. Introduction

Anticipating the demands of future wireless networks, 5G technology and beyond invoke the need for highly efficient, secure, and reliable communications. The design of these networks should meet the emerging applications, virtualization, and artificial intelligence systems that have received great attention recently from countries around the world. Pioneering steps in research and development in wireless communications have been beneficial in directing this technological development. Recognizing the wireless environment as an inherently unsupervised entity, innovative approaches have been enthusiastically explored to mitigate its drawbacks. To achieve the overarching goal, diverse strategies have been deployed, including integrating multiple antennas, the implementation of intricate encoding and decoding algorithms at communication endpoints, and augmentation of network infrastructure with elements like relays. Among them, NOMA is conceived in numerous access schemes. As opposed to Orthogonal Multiple Access, which is used by many wireless communication systems to separate users (such as TDMA [1], a fundamental technique for managing multiple users by allocating distinct time slots for transmission), Non-Orthogonal Multiple Access (NOMA) allows multiple users to take advantage of the same time and frequency resources [1,2]. NOMA works by using some key operations like superposition coding at the transmitter and Successive Interference Cancellation (SIC) at the receiver [2,3,4,5]. SIC is an approach where the receiver first decodes the strongest signal, removes it from the combined signal, and repeats this step until all signals are decoded.

For SIC to perform optimally, accurate Channel State Information (CSI) is essential and provides detailed knowledge of the channel conditions, resulting in sustained communication, even in hard interference conditions [6]. Other important parameters can play an important role in the system performance in general; for example, Liu et al. [7] tested the impact of system parameters, such as power allocation, user ordering, and channel conditions. Yet, NOMA works extremely well in a less challenging environment. However, in the case of channel condition degradation, any communication blockages, including obstacles and adverse weather like, for example, heavy rain, snow, and dense fog, can reduce signal strength and clarity, potentially disrupting the entire system. NOMA alone may not be sufficient for these instances, and it could therefore lead to the development of systems made up of technologies such as MIMO systems, cooperative communications, IRSs, and advanced signal processing techniques for increased resistance to interference, as well as for improved reliability.

NOMA typically operates through line-of-sight (LoS) links, which reduces its coverage area and increases sensitivity to blockage. However, when it comes to these limitations, particularly NLoS conditions or bad channels due to obstacles, or even bad weather, it is vital to seek and integrate supporting technologies. Technologies such as MIMO [8], cooperative communication, and IRSs [9] can be among the methods for optimizing system quality. These technologies allow us to automatically adjust to fluctuating environments, enabling stable and consistent connections, even when LoS is interrupted. Through the application of these new technologies, NOMA networks can become more robust in challenging scenarios and diverse use cases, ultimately making them more effective overall.

A list of specific topics was covered to enhance the signal quality and address the issues caused by non-line-of-sight environments such as Channel Modeling and Characterization [10], Diversity Techniques [11], power control, Efficient Precoding and resource allocation [12], and relaying for a hybrid satellite [13]. Ref. [14] focused on the broader aspects of encoding and decoding algorithms for relay satellite systems, which involve some considerations such as channel coding, modulation, synchronization, and error correction.

**Figure 1:**
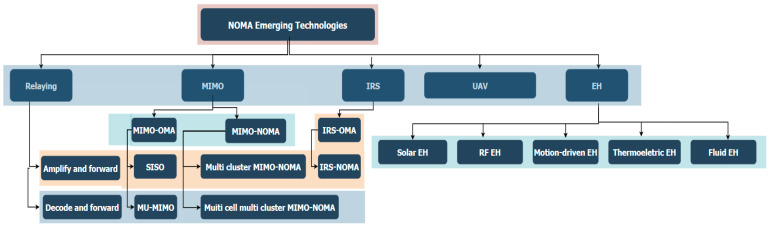
NOMA emerging technologies.

This paper provides a comprehensive and up-to-date review of wireless technologies that are developed specifically for the difficulties faced in NLoS environments within wireless communication. We focus on NOMA-integrated technologies such as MIMO, cooperative NOMA, IRS-NOMA, and UAV-NOMA, which are specifically developed to improve performance in NLoS conditions. Unlike other papers that mainly focused on LoS environments, our review examines thoroughly the impact of NLoS on wireless communication. We thoroughly explore NOMA-integrated technologies, focusing on how they can address NLoS issues and give these technologies the detailed attention they deserve. Figure 1 demonstrates NOMA’s emerging technologies and some other aspects, which will be highlighted.

## 2. NOMA Emerged Technologies

### 2.1. C-NOMA

**Figure 2:**
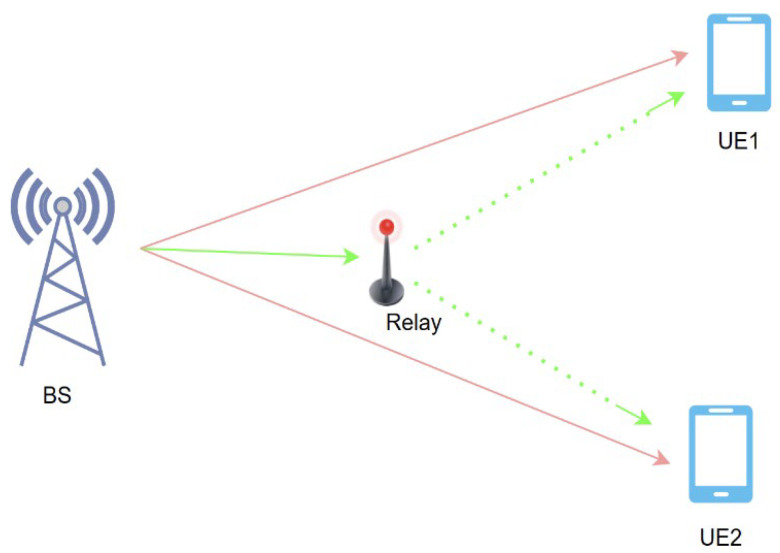
System model for cooperative NOMA.

The integration of Non-Orthogonal Multiple Access in cooperative relaying brings a new dimension where User Equipment (UE) decodes signals from other UE using Successive Interference Cancellation, and serves as a relay to improve the overall performance of the wireless system in NLoS conditions where there is limited coverage and susceptibility to signal blockage [15]. In a collaborative approach, which is explained in [16,17], a slot-based transmission involves users acting as relays, forwarding messages from the base station (BS) to various users within the system at different distances. Multiple slots are used in transmitting messages, with the BS sending a superposed message to all its users initially. It later picks one user, based on channel gains, as a relay for each slot before having the remaining ones forwarded to other users who have not been relayed or serving that role at present. This process continues until the user with the weakest channel is served last, thus optimizing overall system performance. Figure 2 shows an example of a cooperative approach where a relay is used to help both users have their signals forwarded from the base station.

There are several reasons why relaying solutions is important in cooperative systems because it assists in increasing coverage, reliability, power efficiency, and spectral efficiency [18]. In the Amplify and Forward (AF) approach [19,20,21], a relay just scales the received signal from the source node and then transmits it to the destination. As opposed to this, the decode and forward (DF) algorithm [22,23,24] is characterized by a more complicated procedure, including both decoding and re-encoding steps performed by a relay before its transmission to an end-user. This division of relaying strategies emphasizes how diverse approaches are utilized in signal transmission optimization while at the same time bringing into focus sophisticated techniques in cooperative communication systems.

#### 2.1.1. Dynamic Decode and Forward (DDF) NOMA

In this system, the transmitter configures superposition coding and power allocation and then sends the signals. Receivers then dynamically figure out a decoding order based on effective channel gains with which transmission is performed. This scheme demonstrates considerable outage probability gains over the fixed-order NOMA, which is considered conventionally in dynamic-order NOMA operations. Refs. [25,26,27] propose a systematic approach aimed at boosting performance through a cooperative system where the power levels are optimized for each pair of NOMA users to minimize the overall outage probability instead of using a single level for all pairs. This is called the dynamic ordering/selection of receivers and relays that rely on channel conditions regarded as effective, not a fixed protocol.

#### 2.1.2. Dynamic Relay Selection (DRS)-Fixed Power Allocation (FPA) and DRS-Dynamic Power Allocation (DPA)

Dynamic power allocation strategies [28,29] are proposed to optimally allocate power resources among users based on their channel conditions and interference levels, to maximize the overall system sum rate. Additionally, both papers investigate dynamic relay selection mechanisms to choose the best relay for each weak user, considering factors such as channel quality, interference levels, and resource utilization. Furthermore, the papers integrate Coordinated Multi-Point (CoMP) techniques with NOMA to mitigate inter-cell interference and enable coordinated transmission and reception among multiple access points, enhancing overall system performance and resource utilization.

**Figure 3:**
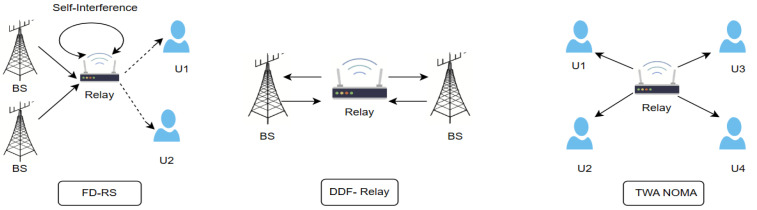
Examples of cooperative relaying schemes.

Similarly, Xie et al. [30] proposed a study of user pairing and power allocation for a SWIPT-enabled NOMA relay system. It presents an optimum user selection mechanism that assists in enabling the users to experience less channel degradation for information decoding at the relay. Then it focuses on both fixed and dynamic strategies for power allocation. Fixed power allocation may guarantee simplicity, yet dynamic power allocation will allow for an increased performance due to the instantaneous conditions of user pairs and the relay energy constraints. The case of dynamic allocation shows that the outcome is significantly better from the aspects of low outage probability and high throughput than from the aspects of static allocation, which in this case helps to address the issues of wireless communications in NLoS environments. Figure 3 shows some examples of cooperative relaying schemes.

#### 2.1.3. Cooperative Relay-Based FD NOMA (FD-NOMA-RS)

The user in FD mode is utilized by the relay in the same fashion where the decode and forward strategy is followed from NOMA users to the base station. The stages of the relay, after receiving the superposed NOMA signals from the users, decode them for the BS and loop interference from its own transmit signal. Analytical results for the outage probability show that the FD scheme demonstrates better performance than the conventional HT and HD relaying. The authors in [31,32] explored the integration of NOMA in uplink and cooperative transmissions, as well as the application of the FD relaying technique. They noted that the net data rate would be higher than that of the conventional half-duplex relaying. On the other hand, FD, together with the loop interference result, should be governed by splitting of power in time or optimal power control algorithms studied in the recent literature to achieve the complete benefit of FD capability in NOMA systems.

#### 2.1.4. Two-Way Relay (TWR)-NOMA

A scenario where two different NOMA user groups are communicating with each other indirectly through relays [33]. These relays use specific relaying techniques called Amplify and Forward (AF), decode and forward (DF), Selection Relaying (SR), and Hybrid Relaying (HR) to help forward the signals between the groups. The base station exchanges information with the users via an assisting relay node using a two-phase TWR protocol integrated with NOMA. In the first phase, the superimposed multi-user signals are transmitted to the relay. The relay then decodes, re-encodes, and broadcasts a combined signal back to the users/base station in the second phase using network coding principles [34,35,36].

### 2.2. MIMO-NOMA

Improving wireless communication performance depends on increasing throughput (bit/s), which is determined by bandwidth and spectral efficiency. Any strategic enhancement of either or both factors results in increased throughput.

Combining MIMO with NOMA offers several advantages, such as improved spectral efficiency, increased system capacity, and enhanced user fairness. By exploiting the spatial diversity of MIMO and the non-orthogonal multiplexing capability of NOMA, the combined MIMO-NOMA approach can effectively serve multiple users simultaneously over the same time–frequency resources. However, there are some significant challenges in realizing the full potential of MIMO-NOMA systems. One major challenge is the increased receiver complexity due to the need for SIC and multi-user detection algorithms [37]. In the Single Input Multiple Output (SIMO) NOMA environment, the use of Successive Interference Cancellation techniques helps remove such computational blocks. On the other hand, things become more complicated in MIMO-NOMA when a substantial number of clusters introduce inter-cluster interference. To address this, cluster design involves a holistic approach through beamforming power allocation and SIC. Beamforming with the additional spatial degrees of freedom and power allocation among users eliminates interference both in terms of signal strength as well as location [38,39,40], thereby aiding MIMO-NOMA in complex design challenges.

**Figure 4:**
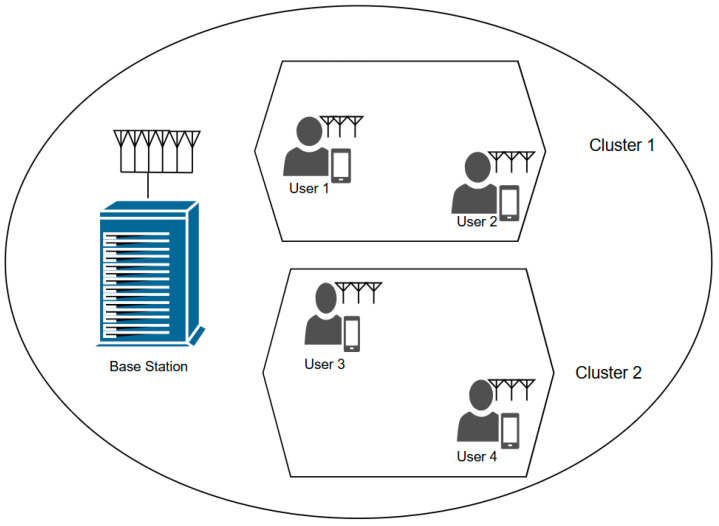
Multi-cluster MIMO-NOMA.

The combined implementation of MIMO and NOMA provides an advantage in acquiring high spectrum efficiency and downlink throughput gains in environments with poor channel conditions, such as those with high interference, deep shadowing, or multipath fading [41]. This solution includes a large number of base stations and user equipment, allowing increasing system capacity with the help of directional communication, made feasible by beamforming signal processing techniques. This capability is particularly beneficial for covert mmWave communications, where the use of directional beamforming can significantly reduce the likelihood of detection by wardens [42]. Prashar and Sood [43] examined the performance of MIMO-NOMA systems in downlink communication scenarios. Their paper explored the benefits of MIMO techniques in NOMA systems, which can leverage directional communication and beamforming to improve system capacity and performance of multi-user directional beamforming since each user receives an orthogonal dedicated beam. In addition, their proposed system showed the capability to reduce interference and optimize the total sum capacity. There are different MIMO types that can be integrated with NOMA to address NLoS issues such as a multi-cluster MIMO as shown in Figure 4 and a multi-cell multi-cluster MIMO.

#### 2.2.1. Multi-Cluster MIMO-NOMA

In a multi-cluster MIMO-NOMA system, the SIC procedure is executed sequentially within each cluster. The unique structure of these clusters, established at the base station, offers a significant advantage by effectively reducing interference among users across different clusters [44]. In this respect, the segmented grouping of users into clusters can be considered a key strength of MIMO-NOMA systems compared with classical SISO−NOMA single antenna. The most emerging research trends in the field of MIMO-NOMA systems at this stage are directional transmission, user grouping algorithm design, and power distribution among users, as well as sequential demodulation algorithms [45,46,47]. Allocation of a majority of users within one cluster, however, complicates decoding and may result in a decrease in the total sum rate. To resolve these challenges, the use of a multi-cluster MIMO-NOMA model is advantageous. In this model, the number of clusters typically matches the number of base station transmitter antennas. Users with multiple receive antennas are grouped into clusters, with power allocation in each cluster following NOMA rules. This approach improves decoding efficiency and enhances overall system performance [48]. This innovative method addresses the trade-offs stemming from large clusters, providing a dynamic balance between network complexity and system sum rate within MIMO-NOMA systems.

#### 2.2.2. Multi-Cell Multi-Cluster MIMO-NOMA

**Figure 5:**
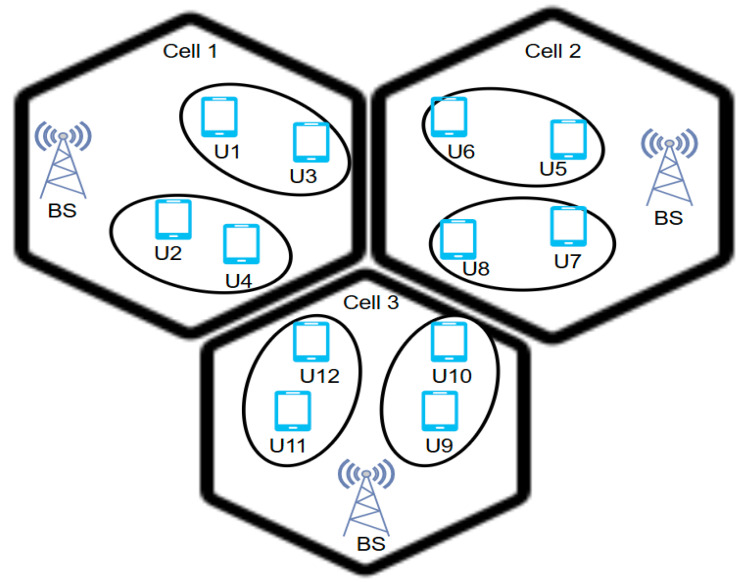
Multi-user multi-cluster MIMO-NOMA.

In the enormous space of multi-cell multi-cluster MIMO-NOMA systems, a serious complication emerges as inter-cell interference for users near boundary cells. This interference not only degrades data rates but also affects fairness, especially for users at the edge of a cell [49]. One major challenge in increasing spectral efficiency within this network is the increased interference due to the use of large antennas associated with the NOMA approach. A possible solution to this problem is the reuse of resources with proper scheduling and optimization techniques. As shown in Figure 5, the downlink transmission of a multi-cell MIMO-NOMA system is illustrated, where the base station (BS) comprises N number of antennas servicing several M clusters. Each M cluster consists of two users, both served by a single antenna from the corresponding base station.

The BS combines messages of these two users that are desired from this M cluster. Then it delivers the aggregated signal with different power allocation coefficients based on a power domain NOMA using a beamforming vector, all in one time and frequency slot. Every user denoted as the kth user also extracts his desired signal and that of another intended for a distant subscriber within the M cluster. The reception process utilizes SIC for identifying and separating the desired signal from each user while simultaneously eliminating inter-cluster interference [50]. By optimizing power allocation and utilizing advanced interference management, this system ensures robust communication, even in challenging NLoS environments.

### 2.3. IRS-NOMA

**Figure 6:**
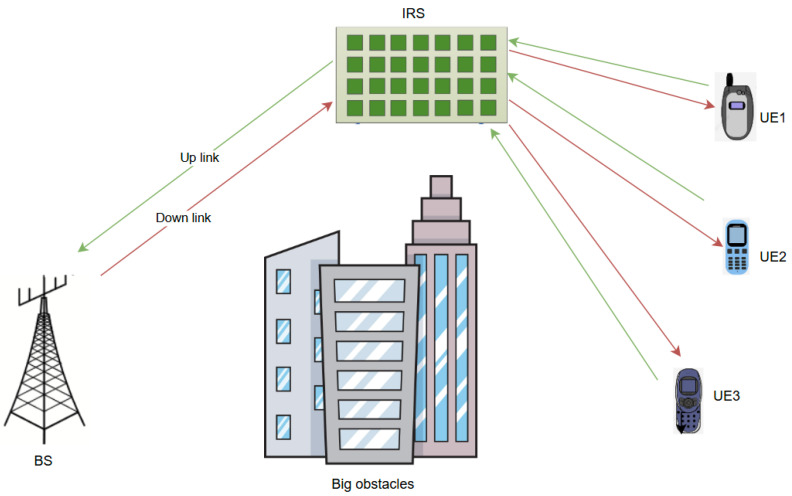
IRS-NOMA.

The introduction of intelligent reflecting surfaces into wireless communication networks has become an innovative approach to overcoming non-line-of-sight challenges and increasing energy efficiency, as well as spectral efficiency. IRSs can modify a unique wireless signal propagation environment that features multiple scattered signals from various reflectors. This reconfiguration guarantees that the incoming signal is reflected to its target user while being rejected by others, as shown in Figure 6.

An IRS is characterized by deployment flexibility and convenience, as installation can be carried out on building facades, walls, or ceilings with little power usage. In particular, the IRS prevents the use of active RF components that are necessary for signal processing and transmission. Structurally, an IRS comprises three layers: a me-ta surface layer made of subwavelength particles in a planar array, a copper layer that prevents the leakage of signal energy, and a circuit board linked to an IRS controller [51].

Traditional NOMA, while effective in enhancing system throughput and user capacity using enabling multiple users to share the same time and frequency resources through power domain multiplexing, is still confined by the existing propagation conditions. It depends solely on the availability and disposition of inherent signal paths and suffers large performance penalties in NLoS situations due to the existence of attenuating and interfering obstacles. On the other hand, IRS-NOMA introduces a transformative approach by integrating the IRS. These programmable surfaces can then change the phase and direction of the electromagnetic waves in a way that they build up new paths with better characteristics. Not only does this capability mitigate the problem associated with signal blockage but also works to increase the signal strength and quality, even in those environments that fall under the NLoS category. By incorporating an IRS, IRS-NOMA extends the flexibility and adaptability of traditional NOMA, providing a more robust and reliable communication framework that can dynamically respond to the spatial characteristics of the environment, thereby significantly improving overall system performance.

In traditional NOMA, channels are typically assigned to users based on the propagation environment, and it does not require tuning. On the other hand, the IRS provides a disruptive capability through the adjustment of phase shifts based on its phase-shifting function [52]. This dynamic adaptation gives a significant edge to IRS-NOMA over NOMA. By tuning the phase shifts in the IRS, a significant difference in users’ channel gains is obtained, making NOMA highly applicable. The combination of the IRS and NOMA has attracted much attention due to the numerous advantages that it provides. It certainly not only copes with issues specific to NLoS surroundings but also opens new horizons in the field of wireless communication, improving system efficiency and starting a thematic direction [53]. The following lines of the survey cover innovative strategies for configuring the IRS, both statically and dynamically, to maximize gains in IRS-NOMA systems over OMA systems. It also explores how the IRS can improve wireless power transfer, radar signal quality, and detection accuracy. Additionally, it analyzes various IRS operation modes to optimize performance for different applications.

#### 2.3.1. Static and Dynamic IRS Configurations In the Context of Broadcast Channels (BCs)

In a static configuration, the reflection coefficients of intelligent reflective surfaces remain stable throughout transmission and, therefore, position the IRS-enabled Broadcast Channel with its conventional equivalent. The process of unlocking the capacity region for RIS-enhanced BC under this static setting requires a thorough analysis of all possible reflection coefficients. The scale of this investigation is closely connected to the number and resolution that can be applied to control the phases of these IRS elements. Additionally, the feasible rate regions for both TDMA and FDMA modalities can be derived from similarly realizable rate vectors of each specific scheme. However, the IRS-based dynamic configuration is characterized by reconfiguring its reflection coefficients N times in a transmission instance. The challenging issue arises when identifying the capability region, as identification for dynamic IRS configuration grows exponentially with respect to N, along with an increase in set accommodating all possible choices of reflection coefficients. In a more complex environment, the scenario is considered where N approaches infinity as a way to set an upper bound of performance. In such an environment, time-sharing becomes pivotal and scattered among all potential reflection coefficients [54,55].

The convex hull of all achievable capacity regions for the static IRS takes form as a capacity region for N approaching infinity. It must be emphasized that this method fails to consider the time needed for configuration and update of the IRS, assuming smooth real-time updates. The energetic IRS design is announced to achieve the potential of scrutinized IRS-enabled multi-user BC, especially as N tends towards a value for infinity [56,57].

#### 2.3.2. IRS-Assistant Wireless Power Transfer

Beyond wireless communications, the RIS is also being investigated as a transformative solution to significantly boost the efficiency of radio frequency (RF) wireless power transfer (WPT) systems. Important work by Shi et al. [58] highlighted the pros and cons of achieving wireless energy harvest in RIS-based cell-free MIMO architectures. The potential of the RIS to enhance WPT has been experimentally validated by Tran et al. [59] using a 5.8 GHz 1-bit RIS prototype. While focusing on WPT, the concepts are relevant to addressing NLoS issues in wireless communication. By using the IRS, signals can be intelligently redirected and optimized, improving signal strength and reliability in NLoS environments. Experimental results showed that the RIS can achieve around a 20 dB improvement in received power and a much higher power transfer efficiency compared with traditional systems, indicating its potential to transform NLoS wireless communication scenarios as well. This groundbreaking work has opened up an exciting research front to examine IRS-aided SWIPT (Simultaneous Wireless Information Power Transfer) systems [60,61], where the IRS can coherently focus the incident RF signals toward intended receivers for efficient information decoding while simultaneously maximizing the energy harvesting capabilities over large areas.

#### 2.3.3. IRS-Aided Wireless Radar

The unique ability of the IRS to manipulate electromagnetic wavefronts has opened up new possibilities for integrating radar and communication functionalities on a shared platform. Ref. [62] proposed an IRS-assisted spectrum-sharing framework for MIMO radar to coexist with a multi-user MISO communication system, while Yan et al. [63] developed a dual-function IRS-aided system capable of simultaneous radar sensing and wireless communications, by exploiting the wavefront transformation capabilities of the IRS. To enhance radar target detection in such integrated architectures, Xiao et al. [64] introduced efficient constant-modulus waveform designs tailored for IRS-aided dual-functional radar operation. For NLoS scenarios, Aubry et al. [65] demonstrated how an RIS can tackle the radar sensing challenges by establishing an intelligent reflecting surface that bridges the propagation paths. In the millimeter-wave regime, Ref. [66] proposed an IRS-aided MIMO radar architecture with adaptive beamforming for multi-target localization and tracking.

Furthermore, Ref. [67] developed a joint optimization framework for waveform design at the radar transmitter and passive beamforming configuration at the IRS to maximize the radar-communication performance trade-off, which demonstrated the IRS’s potential to enhance NLoS applications.

#### 2.3.4. IRS Operation Modes

An IRS operates in various modes, each with an advantage for propagating signals in NLoS. The reflective mode uses the control so that it redirects the incoming signals to desired directions, creating the virtual LoS path that goes through obstacles. Transmissive mode though not very popular allows the IRS to transmit the signals, making it favorable for the coverage of the network and the objects that may suffer from the weak signal penetration. The hybrid mode has both reflectivity and transmissivity and can change either functionality based on the environment it is in. In addition, IRS technology has a feature of intelligent beamforming [68], which ensures that the phase and amplitude of reflected signals are accurately concentrated on a specific area. Such operation modes help to improve the signal strength, reliability, and coverage of NLoS applications, which may provide a promising perspective for the RIS to achieve the potential of emerging wireless communication and sensing systems.

The basic operating modes of an IRS are discussed in Table 1. Instead of reflecting the electromagnetic wave symmetrically according to Snell’s law, the RIS surface is divided into many closely packed components (supercells of scattering particles). Each elements meta-surface element imparts a suitable phase shift, allowing the EM wave to be tuned and reflected at any desired angle [69]. Ideally, if the phase shift of each meta-surface element can be precisely calibrated to any value, the reflected beam can be generated at any arbitrary angle.

### 2.4. UAV-Enabled NOMA

Unmanned Aerial Vehicles (UAVs), popularly known as drones, are aircraft systems that fly in the air without a human onboard and in the cockpit [70]. Drones can be controlled remotely one by one or fly autonomously based on preconfigured flight plans or more sophisticated dynamic automation. They are being increasingly integrated into NOMA systems in wireless communications, particularly in an NLoS scenario [71]. Rolly et al. [72] proposed a detailed study on the use of UAVs in the applications of NOMA and NLoS scenarios. They outline how UAVs could be used to extend the number of stations and increase the speed of data delivery in NLoS areas by taking advantage of their mobility and flexibility. Nonetheless, they offer no technical solutions.

A study [73] revealed that combining OTDOA 5G positioning algorithms with UAV sensors can provide accurate UAV positioning even in urban NLoS environments. This will help NOMA in such situations to help UAV-aided communications be more reliable. They verified the outcome of the proposed method through the simulations they carried out. On the other hand, Chen et al. [74] studied the integration of reflective surfaces to help NLoS UV communications from a UAV site. They demonstrate via experiments that the deployment of reflectors strategically can deal with the challenges of path loss and background noise in NLoS UV links. This may be a possible way to improve UAV-based NOMA in NLoS conditions as well. In [75,76], authors combined NOMA with UAV-aided DF relaying to enhance cell-edge user performance in NLoS situations. Both the UAV height and resource allocation are optimized to obtain the highest sum rate. Theoretical analysis and simulations verify that NOMA provides better performance than OMA in this case, and the joint optimization approaches seem especially promising for enhancing NLoS NOMA communications. Furthermore, Ref. [77] focused on experimental studies of NLoS optical communication between UAVs and ground stations using visible light wavelengths (450 nm and 510 nm), which provide valuable insights for designing NLOS optical communication systems between UAVs and ground stations, such as the maximum achievable baseline distances for stable communication at different UAV heights and wavelengths. A comparison table between different NLoS NOMA approaches is shown in Table 2.

### 2.5. Energy Harvesting for Sustainable NOMA

While energy harvesting NOMA (EH-NOMA) may not be a primary solution for addressing non-line-of-sight (NLoS) challenges, it shows exciting possibilities for future NOMA deployments. By integrating energy harvesting capabilities into NOMA systems, EH-NOMA can potentially extend the coverage and operational range of NOMA networks, allowing for more flexible and reliable setups. It is worth highlighting the key reasons for the inclusion of the energy harvesting investigation in our work as follows:

Self-sustaining networks: in NLoS environments, energy harvesting allows for the deployment of self-sustaining communication nodes, which is crucial for remote areas, disaster recovery scenarios, and IoT applications.

Enhancing performance and reliability: integrating energy harvesting with NOMA and IRS technologies improves overall performance and reliability. For example, energy-harvesting nodes can continuously power IRS elements, ensuring consistent signal enhancement and coverage in NLoS conditions.

Alignment with future trends: including energy harvesting aligns our work with the vision of future wireless networks, which aim to be adaptive, energy efficient, and capable of operating in diverse environments.

Energy harvesting also refers to the process where devices are equipped with mechanisms that enable them to continue running on natural or artificial sources of energy. This could mean the end of wireless networks that are always down or almost always down, as they would be self-sustaining networks. This is particularly beneficial in NLoS environments where power infrastructure may be limited or inaccessible. The arrival of energy harvesting in wireless networks is surely going to produce revolutionary changes that would include benefits such as reducing the dependency on regular power sources, lower carbon footprint, unlimited mobility through battery-free recharges, and installation of networks everywhere, including remote parts of rural areas or concrete buildings down to the human body [78,79]. In NLoS scenarios, obstructions can cause signal attenuation and power loss, limiting the effective communication distance. However, by harvesting energy from ambient sources, EH-NOMA devices can maintain their power levels and continue operating, even in the presence of obstacles [80]. The literature is full of various advantages of wireless sensors and possibilities for energy harvesting technologies. For example, a study in [81] investigated how energy harvesting can enhance 5G wireless connectivity by overcoming battery lifetime limitations and cutting down operational costs/energy footprints. C-NOMA schemes have explored energy harvesting relays in [82] to improve outage performance, where the relays first harvest energy from received signals before cooperatively forwarding information to enhance coverage.

Novel interference-aided RF energy harvesting protocols have been proposed in [83] for cooperative NOMA networks. These protocols intelligently recycle interference signals as a source for energy collection to improve energy efficiency. For IoT and low-power wireless sensor applications, harvesting ambient energy from sources like light, vibrations, and fluid flows presents attractive self-sustaining solutions, as discussed in [84]. In addition to using photovoltaic energy harvesting systems equipped with tracking light sensors [85], a solar panel alignment can be dynamically optimized to have the most exposed solar rays, thereby improving the conversion efficiency of solar energy. Energy hybrids equipped with the IoT capability to recycle waste derived from multiple ambient sources, such as water flow in rivers and pipelines, have been developed successfully in the latest stage of technology [86]. To take on this challenge, Mu and Sun [87] came up with exact mathematical models of non-linear energy harvesting that are based on sum energy maximization algorithms. By addressing the challenges of trajectory design, energy allocation, and non-linear energy harvesting through joint optimization, the solutions proposed in this paper aim to maximize the sum of energy transferred to ground nodes and optimize the overall RF charging performance over large areas served by UAV-enabled wireless power transfer networks.

#### 2.5.1. Solar Energy Harvesting

In the past twenty years, solar energy harvesting technology has made great strides, moving from space programs to everyday use. By ensuring a continuous power supply, solar energy harvesting can help maintain communication links in NLoS situations caused by physical obstructions. Since this technology depends largely on the performance of PV panels and the conversion efficiency, various Maximum Power Point Tracking (MPPT) methods have been developed to enhance solar power conversion. However, unstable input voltage to the DC-DC converter remains a challenge, requiring efficient design. Additionally, because energy from the environment is not always constant and even might be irregular, it is vital to create reliable energy storage methods.

Losses that take place in solar cells play an important role during the whole process of transformation from solar energy to electric energy, determining efficiency [88,89,90]. Apart from efficiency factors, the current solar cell technologies have risks such as hotspot formation in parts of the module that tend to threaten the integrity of a given material. The need to address these challenges and optimize solar–electric conversion processes will always be focal points in improving both the efficiency and sustainability of harvesting technologies powered by solar.

#### 2.5.2. Radio Frequency (RF) Energy Harvesting

**Figure 7:**
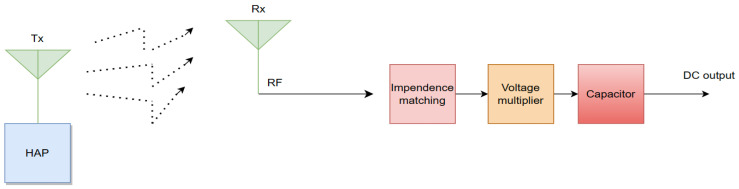
RF energy harvesting block diagram.

Energy harvesting can utilize ambient RF signals that are usually available in urban and densely populated areas. This technology will enable NOMA devices to continue operating when there are no direct power sources available. RF energy harvesting will help NOMA systems maintain power levels and continue with communication, even in NLoS circumstances. Figure 7 shows the block diagram of the RF energy harvester consisting of a Hybrid Access Point and an energy harvesting device. The frequencies of RF energy transmission typically range between 300 MHz and 300 GHz, with antennas working within this spectrum either individually or combined [91]. The propagation or beamforming can be omnidirectional or directional, depending on the application of the energy broadcast. Omnidirectional signals are good for low-energy broadcasts, while directional signals often obtained from the aerial array are optimal options for high-intensity point-to-point energy transfer processes [92].

Even though the RF energy harvesting systems are comparatively immune to weather, they do have limitations in terms of low power density and increased sensitivity. Some important factors affecting the efficiency of RF energy harvesters are input power level, frequency band, impedance matching point, voltage multiplier circuitry, and load resistance type [93]. Hence, optimizing designs related to RF energy harvesters requires careful evaluation of such parameters.

#### 2.5.3. Motion-Driven Energy Harvesting

This technology can harvest power from vibrations, motions, or human activity. It can be used in mobile or wearable NO-MA devices, where conventional power sources are interrupted or unavailable. The idea of motion energy harvesting can complement NOMA systems and help them to keep working in dynamic and challenging environments [94]. For low-frequency environmental vibrations, Ref. [95] proposed an electromagnetic harvesting mechanism to convert bidirectional vibrations into unidirectional rotations to improve energy transduction efficiency. Focusing on human motion as the energy source, Choi et al. [96] provided a comprehensive review of recent advances in nanogenerator technologies that can directly harvest biomechanical energy from body movements like walking, breathing, and muscle contractions. Such human-powered generators can enable self-powering smart sensors and self-charging personal electronics [97]. Specific to wearable applications, Cai et al. [98] examined the latest human motion excited energy harvesting systems, discussing their potential to realize the perpetual, battery-free operation of wearable devices.

**Figure 8:**
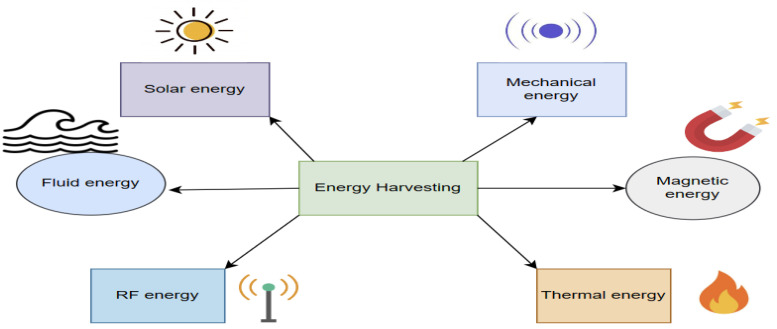
The sources of the harvested energy.

From a materials perspective, Ref. [99] conducted a comparative analysis between piezoelectric and triboelectric energy harvesters for impact-driven scenarios, evaluating their respective performance characteristics and suitability for different use cases. Overall, motion-driven energy harvesting offers a promising approach to achieving energy autonomy and sustainability in NLoS situations, and can maintain communication links for wearable NOMA technologies. Figure 8 shows some sources of different energies that can be harvested.

#### 2.5.4. Thermoelectric Energy Harvesting

Thermoelectric generators translate heat gradients into electrical energy. This technology can be implemented into the NOMA systems to effectively harvest waste heat from electronics or industrial processes. Supplementing conventional power supplies, thermoelectric energy harvesting could improve the robustness of NOMA networks in NLoS environments, where conventional power infrastructure may be insufficient.

Extensive research has been conducted to optimize thermoelectric materials and characterization techniques, aiming to maximize the efficiency and performance of thermoelectric energy harvesting systems. These studies have focused on various aspects, including material properties and device configurations [100], high-efficiency power converter design tailored for thermoelectric energy harvesters [101], and integrated cold-start circuits specifically for thermoelectric harvester operating conditions and ultra-low-power applications [102].

From a systems perspective, Lenz et al. [103] provided a comprehensive assessment of using thermoelectric generators to power self-sustainable wireless sensor nodes by harvesting energy from heat sources. The application of thermoelectric harvesting on RF power amplifiers was explored in [104], demonstrating its feasibility for recovering waste heat. An integrated wireless energy and thermoelectric energy harvesting system is proposed in [105] to power low-power passive sensor networks. Taking integration one step further, Sugiura et al. [106] presented a fully integrated thermoelectric energy harvesting solution using chip-scale thermoelectric generators, paving the way for highly compact self-powered systems. Overall, by providing a continuous power supply from ambient heat sources, thermoelectric harvesters ensure that NOMA devices maintain reliable operation, even in the presence of physical obstructions and challenging conditions, thus enhancing the system’s resilience in NLoS environments.

#### 2.5.5. Fluid Energy Harvesting

Harnessing energy from fluid flows like ocean waves, rivers, and pipelines has attracted significant research interest in powering autonomous devices and wireless sensors. This technology can offer continuous and renewable power sources for NOMA devices and is particularly suitable for employing energy sources in places that are hard to access or where other sources of power are limited. This technology will prevent NOMA systems from losing their functionality in difficult NLoS situations.

Water flow energy harvesting through piezoelectric materials has attracted considerable interest and is regarded as a power supply method of electronics. There are significant efforts made by investigators to work with this technology’s different parameters to enhance the technology’s efficiency and usability. Some of the research opportunities are designing efficient oscillation structures to maximize energy conversion, selecting high-performance piezoelectric materials, developing intelligent power management circuits for energy storage and distribution, and identifying potential applications where this technology can have the greatest impact. An example of an integrated system for wave energy harvesting is presented in [107], detailing the design of a buoy-based generator to extract power from ocean surface waves. For low-power IoT sensors, Kim [108] developed a wireless energy harvesting IC that can operate by scavenging energy from fluid flows.

Focusing on impact-based flows, Azangbebil et al. [109] investigated a soft piezoelectric energy harvester that uses magnetorheological fluids to improve energy conversion from fluid impacts. For residential water pipelines, [110] evaluated the feasibility of using in-pipe turbines as energy harvesting devices, particularly in tropical climates. In the context of energy harvesting transmitters, Koukoutsidis [111] introduced a fluid reservoir model to analyze the information age. The authors in [112] conducted a case study on deploying Wells turbines in the Black Sea to harvest wave energy, sizing a 5 kW turbine system. A hybrid resonant wave energy-harvesting buoy designed for powering marine sensor applications is presented in [113]. Overall, fluid flow energy harvesting shows promise in realizing self-powered systems by tapping into the kinetic energy present in various natural and man-made fluid flows.

#### 2.5.6. EH Challenges for NLoS

The efficacy of energy harvesting in NLoS environments faces significant obstacles. Firstly, obstructions that block the line-of-sight path can also adversely impact the energy harvesting efficiency. Solar panels or RF energy harvesters may experience reduced power generation due to shadowing or signal attenuation, limiting the available energy for NOMA operations. In addition, the inherent power constraints of energy harvesting systems could pose challenges for reliable NOMA transmissions in NLoS scenarios [114].

Fluid, thermoelectric, and motion-driven EH systems, while not reliant on clear paths, still face challenges in NLoS environments. For instance, fluid flow may be disrupted, thermal gradients may be less stable, and mechanical vibrations may be inconsistent. NOMA’s principle of superimposing multiple users’ signals requires careful power allocation and interference management, which may be hindered by the intermittent and fluctuating nature of harvested energy sources. Thus, maintaining the required Signal-to-Noise ratios (SNRs) for SIC and user separation could become increasingly difficult in NLoS conditions. Furthermore, the presence of obstacles can disrupt the Channel State Information (CSI) acquisition process, which is crucial for optimal user clustering and power allocation in NOMA systems [115]. These challenges highlight the need for innovative solutions that seamlessly integrate EH-NOMA with other technologies, such as the RIS or relaying mechanisms, to mitigate the adverse effects of NLoS propagation and ensure robust and efficient NOMA performance in obstructed environments. Table 3 compares various energy harvesting techniques, offering a detailed analysis with a particular focus on NOMA integration and applicability in NLoS environments.

## 3. Current Challenges and Future Directions

NOMA faces several challenges, especially in managing interference among users who share the same resources. Techniques like SIC are essential but hard to implement on a large scale. Additionally, optimizing resource allocation, such as power and bandwidth, is more complex compared with traditional systems like OMA [116,117]. When NOMA is combined with advanced technologies like MIMO, cooperative relaying, IRSs, and UAVs, the challenges increase, particularly in non-line-of-sight (NLoS) situations. The dynamic nature of these technologies complicates interference management and real-time resource allocation and also raises security concerns [118].

Looking ahead, using machine learning and AI can help manage interference more dynamically, optimizing SIC processes and improving system reliability [119]. Developing robust security measures for NOMA’s unique signal structure is crucial to protect against threats [120,121]. Real-world testing and standardization are necessary to ensure these technologies work well together and perform optimally in different environments [122,123]. These advancements will enhance communication reliability and efficiency in NLoS environments, making wireless networks more advanced and efficient.

## 4. Conclusions

In this paper, we have provided a broad review of salient modern approaches that position themselves as potential remedies for the challenges experienced in dense network layouts and non-line-of-sight architectures. We have considered both Orthogonal Multiple Access (OMA) and Non-Orthogonal Multiple Access (NOMA), whereby we have stressed the advantages of NOMA implementations for different communication networks. In addition, we have also investigated current trends in cooperative NOMA, MIMO-NOMA, IRS-NOMA, UAV-enabled NOMA, and energy harvesting NOMA. We have examined how combining NOMA with cooperative relaying and integrating an IRS can improve energy efficiency and coverage. Various performance analyses, such as capacity regions of fixed and dynamic IRS configurations, have also been highlighted. The review work has highlighted how bringing essential features of five different technologies under one umbrella addresses the NLoS problems in wireless communications. Last, but not least, it has illuminated potential research paths, opening the door for formulating practical problem solving in future 6G network environments.

## Figures and Tables

**Table 1:** IRS operation modes.

Operation Mode	Characteristics and Functions	Applications
Reflection	Consider it as the mirror for radio signals. The signal is amplified, which in turn increases the coverage and eliminates interference.	Outdoor environments (focusing signals on specific areas)
Refraction	This mode works like a glass that straightens the route of radio signals.	Outdoor to indoor scenarios (directing signals into specific building areas)
Absorption	Specific bands or frequencies are preferably utilized to essentially block additional noises.	Privacy and information security (indoors/outdoors)
Backscattering	Scattering radios over a wide area, which makes it suitable for regions where signals appear to be missing.	Wide-angle blind spot coverage
Transmitting	RIS will no longer reflect but instead become part of the transmitter itself (emitting, shaping, and directing the outgoing radio waves).	Dynamic Meta-surface Antennas (DMA)
Receiving	An IRS panel acts as both a transmitter and a decoder of radio signals at the same time.	IRS-assisted backscatter communication

**Table 2:** UAV-NOMA significance in NLoS.

Approach for NLoS NOMA	Potential
5G OTDOA positioning + UAV sensors for reliable NLoS operation [71]	- Accurate UAV positioning in urban NLoS environments - Facilitate reliable UAV-aided NOMA communications
UV NLoS link enhancement using passive reflectors [72]	- Reduce path loss and background noise in NLoS UV links - Applicable to UAV-based NLoS NOMA
NOMA + decode and forward UAV relay with height and resource optimization; IRS-aided NOMA UAV relay considering NLoS direct paths [73,74]	- Tackle NLoS issues by jointly optimizing UAV parameters (height, trajectory) and communication resources (power, phase shift) - NOMA gain confirmed by theoretical analysis and simulations
Experimental study of NLoS visible light range communication between UAV and ground [75]	- Feasibility of NLoS optical communication (visible light) between UAV and ground

**Table 3:** Comparative analysis of energy harvesting technologies.

Energy Harvesting Type	Concept	Advantages	Potential for NOMA	Potential for NLoS
Solar Energy Harvesting	Conversion of solar energy into electrical energy using photovoltaic (PV) panels	-Renewable and plentiful energy source -Mature technology	Useful for stationary NOMA nodes in outdoor environments	Limited: success depends on sunlight obstructions
Radio Frequency Energy Harvesting	Harvesting energy from ambient RF signals and broadcast sources	-Remote energy supply -Useful for inaccessible areas	Excellent for urban NOMA setups with abundant RF signals	High: can harvest energy from RF signals in urban environments
Motion-driven Energy Harvesting	Scavenging energy from ambient vibrations and human motion	-Sustainable source from everyday activities -Suitable for wearable devices	Suitable for wearable NOMA devices or nodes with user interaction	Moderate: depends on movement and vibrations
Thermoelectric Energy Harvesting	Converting heat and temperature gradients into electrical energy using thermoelectric materials	-Solid-state technology -No moving parts -Compatible with IoT	Applicable for NOMA nodes in environments with heat differentials	Low: best suited for consistent temperature gradient environments
Fluid Energy Harvesting	Extracting energy from fluid flows like ocean waves, rivers, and pipelines	-Significant power in fluid-rich environments	Ideal for NOMA nodes located near or in water bodies or airflow paths	Moderate: useful in areas with accessible fluid flows but not ideal for typical NLoS scenarios

## Data Availability

No new data were created or analyzed in this study.
